# The Environmental Dependence of Inbreeding Depression in a Wild Bird Population

**DOI:** 10.1371/journal.pone.0001027

**Published:** 2007-10-10

**Authors:** Marta Szulkin, Ben C. Sheldon

**Affiliations:** Department of Zoology, Edward Grey Institute, University of Oxford, Oxford, United Kingdom; Ecole Normale Supérieure de Lyon, France

## Abstract

**Background:**

Inbreeding depression occurs when the offspring produced as a result of matings between relatives show reduced fitness, and is generally understood as a consequence of the elevated expression of deleterious recessive alleles. How inbreeding depression varies across environments is of importance for the evolution of inbreeding avoidance behaviour, and for understanding extinction risks in small populations. However, inbreeding-by-environment (I×E) interactions have rarely been investigated in wild populations.

**Methodology/Principal Findings:**

We analysed 41 years of breeding events from a wild great tit (*Parus major*) population and used 11 measures of the environment to categorise environments as relatively good or poor, testing whether these measures influenced inbreeding depression. Although inbreeding always, and environmental quality often, significantly affected reproductive success, there was little evidence for statistically significant I×E interactions at the level of individual analyses. However, point estimates of the effect of the environment on inbreeding depression were sometimes considerable, and we show that variation in the magnitude of the I×E interaction across environments is consistent with the expectation that this interaction is more marked across environmental axes with a closer link to overall fitness, with the environmental dependence of inbreeding depression being elevated under such conditions. Hence, our analyses provide evidence for an environmental dependence of the inbreeding×environment interaction: effectively an I×E×E.

**Conclusions/Significance:**

Overall, our analyses suggest that I×E interactions may be substantial in wild populations, when measured across relevant environmental contrasts, although their detection for single traits may require very large samples, or high rates of inbreeding.

## Introduction

Inbreeding depression, caused by the expression of deleterious recessive alleles [1], [2], reduces the fitness of homozygous individuals relative to outbred members of the same population. It is assumed that inbreeding depression is mainly caused by dominance effects–i.e. the expression of recessive deleterious alleles, and not because of a specific advantage of heterozygotes (the overdominance hypothesis) [1], [3]. Thus, the strength of inbreeding depression will depend on the genetic load carried by a population. As a consequence, inbreeding depression may not always be visible in inbred individuals, and even within populations it may be environmentally-dependent [4], [5]. Interactions between the inbreeding coefficient and an environmental variable on a fitness-related trait (I×E interactions) indicate that, depending on the quality of the environment, inbreeding depression is variable in magnitude. In addition, however, environments differ in their relevance to overall fitness: some environmental factors may strongly influence fitness, whereas others may have only a weak influence on fitness. Evidence from comparisons of the magnitude of dominance variance across characters suggests that this component of genetic variance is larger for traits with a closer link to fitness, such as life-history characters [6]. Given that inbreeding depression is more marked for such characters, we should expect that the effect of the environment on inbreeding depression will be greater across environmental axes that themselves explain a greater proportion of fitness variation.

Evidence for I×E interactions is accumulating: although some studies have not found evidence for I×E interaction, a recent review by Armbruster and Reed [4] showed that overall, inbreeding depression is greater under stressful conditions. Genotype-by environment interactions for fitness have been reported in many broader contexts [e.g. 7], [8], but are of particular interest in inbreeding because they may contribute importantly to the persistence of small populations. The fact that some deleterious alleles will only be expressed under some environmental conditions, but not others, renders deleterious allele purging difficult [4], [9] and can have far-reaching consequences for captive species management [10] and populations with limited effective size, threatened by extinction.

Studies of I×E interaction in animals have mostly focused on invertebrates in a laboratory, or enclosure, setting [5], [10], [11], [12]. There is a paucity of studies of populations living in their natural environment, and among vertebrates such as birds and mammals in any environment. This is likely to be due to (1) the difficulty of maintaining pedigrees, needed to estimate inbreeding coefficients, in natural populations, and (2) the occurrence of close inbreeding in the wild often being rather low [e.g. 13], [14], yielding very small sample sizes of highly inbred individuals.

Recently, studies inferring inbreeding using genetic marker-based estimates of genetic diversity in wild populations of spiders and marmots showed that lowered heterozygosity may interact with the environment to result in poorer fitness prospects in harsh environments [15]–[17]. However, because heterozygosity does not necessarily reflect inbreeding [18]–[20], further insight into pedigreed populations is needed to understand the relationship between inbreeding depression and its interaction with the environment.

To our knowledge, only three pedigree-based studies [13], [21], [22] have investigated I×E interactions in wild vertebrate populations; these yielded mixed evidence for the occurrence of environmentally-dependent inbreeding depression. Keller et al. [21] and Marr et al. [22] found significant I×E interactions for small and insular populations of cactus finch *Geospiza scandens* and song sparrow *Melospiza melodia*, respectively. In contrast, Kruuk et al. [13], found no evidence for I×E interaction in a larger, open population of collared flycatchers *Ficedula albicollis*. The aim of the present study was to investigate I×E interactions in a wild bird population open to substantial immigration (the great tit *Parus major* studied at Wytham, near Oxford) using a pedigree constructed over 47 years. We used a wide array of environmental cues, encompassing both individual-specific ecological information and cohort-specific estimates of environmental factors, juvenile survival and phenotypic variance, and sought to relate these to the magnitude of inbreeding depression in this population.

## Results

We identified eleven environmental axes, which were hypothesised to affect offspring recruitment-the chosen measure of fitness-to a varying degree (Table 1); all axes were subsequently tested for their interaction with inbreeding. The 58 breeding events where offspring had an inbreeding coefficient *f*≥0.125 occurred in roughly equal proportions in good and poor environments, and there was no evidence that the proportion of inbreeding events detected was related to the quality (good/poor) of the environmental axes (for all eleven measures of environmental quality, we ran a GLM with binomial errors to test the effect of each environmental variable on the proportion of inbreeding events in good and bad environments). The only environmental axis where there was imbalance in terms of the number of inbreeding events in good and poor environments was maternal age, where 22 inbred and 2338 outbred matings were found among first year breeders, and 36 inbred versus 2106 outbred matings were encountered in females older than one year of age. However, this imbalance was not significant after correcting for multiple testing.

**Table 1:** Environmental axes and predictions as to their potential impact on fitness, measured in terms of recruitment success in the great tit.

Environmental axis	Predicted association	Level of analysis	N	% Variance explained
1. Yearly population density of breeding events	Years with higher breeding density are detrimental to offspring survival as there is greater competition for resources.	cohort	41	0
2. Local oak density	Nestboxes with oak trees *Quercus spp.* in close proximity (<50 m.) should benefit from better caterpillar provisioning at the nest, enabling offspring to fledge in better condition.	nestbox	945	0
3. Female parental age (1^st^ year or older)	Older parents are more experienced in raising young and have higher reproductive success.	breeding event	4502	0.1
4. Male parental age (1^st^ year or older)	Older parents are more experienced in raising young and have higher reproductive success.	breeding event	4482	0.4
5. Local population density of breeding events	High breeding density is detrimental to offspring survival as there is greater competition for resources (food). The local density of breeding events is determined using breeding event territory size, determined for each breeding event independently [34].	breeding event	4449	1.1
6. Nestbox distance from forest edge	Nestboxes situated close to forest edges have lowered reproductive success.	nestbox	951	1.1
7. Lag between caterpillar peak and hatching peak	Small lag between average caterpillar peak and population average hatching peak means that in those years, most birds breed too late relative to the optimum breeding date [35].	cohort	32	7.1
8. Yearly quality in fledging mass	Years where individuals on average leave the nest in poorer condition induce lower rates of survival and recruitment to the population in the following year.	cohort	41	14.2
9. Winter beech mast abundance	Beech mast is a valuable food resource for great tits and determines their over-winter survival.	cohort	41	31.4
10. Phenotypic coefficient of variation in recruitment	Inbreeding depression should be most pronounced in years where environmental conditions enhance the variability of fitness traits (D. Waller, pers. comm.).	cohort	41	76.4
11. Yearly quality in recruitment	By definition, yearly average recruitment explains all variance in yearly recruitment.	cohort	41	100

The proportion of variance in recruitment explained by each environmental axis was measured by fitting a linear regression of each environmental axis on recruitment at the appropriate level (cohort, nestbox or breeding event level), and is presented in the far-right column.

**Figure 1:**
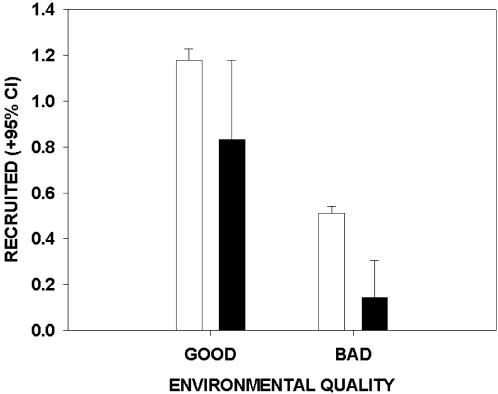
Mean number of individuals from outbred and inbred broods of great tits that recruited in good and bad environments. The reproductive success of outbred (*f* = 0.0) and inbred (*f* = 0.25) broods are represented by white and black bars, respectively. Environmental quality is here defined in terms of each year's mean recruitment success relative to the overall median of all yearly values of recruitment (error bars: 95% CI).

There was a significant effect of inbreeding on recruitment across all eleven environmental axes, but only one inbreeding by environment interaction was found significant (inbreeding * yearly quality of fledging mass; Table 2). The environmental axis that had the strongest effect on recruitment was by definition the annual quality of recruitment, which reflects the overall summation of different environmental factors influencing survival, and thus recruitment. Interestingly, although its interaction with inbreeding was non-significant, we observed a 3.5 fold increase in lethal equivalents in the poor environment relative to the good environment for this environmental axis, with values of B = 5.28 versus B = 1.48 in the poor and good environments, respectively. At the recruitment stage, individuals inbred at *f* = 0.25 had a performance of 71% relative to outbred individuals in a good environment, but only 28% in a poor environment (Figure 1). We suggest that the large confidence intervals around the fitness estimates for the relatively small groups of highly inbred birds (Figure 1) renders the apparent difference in the strength of inbreeding depression between the environments non-significant (Table 2).

**Table 2:** Effect of inbreeding, the environment (measured with eleven environmental axes) and their interaction on recruitment in the great tit.

	Environmental axis	Population density	Local oak density	Female age	Male age	Local population density	Distance to edge	Caterpillar lag	Yearly quality in fledging mass	Winter beech mast	Phenotypic CV	Yearly quality in recruitment
		n = 4365	n = 4322	n = 4345	n = 4325	n = 4304	n = 4340	n = 3711	n = 4365	n = 4365	n = 4365	n = 4365
Inbreeding	Wald	5.54	7.78	6.95	7.57	7.58	7.66	5.43	7.75	5.58	6.81	8.05
	P	0.019*	0.005**	0.008**	0.006**	0.006**	0.006**	0.020*	0.005**	0.018*	0.009**	0.005**
	parameter estimate (S.E.)	−1.93 (0.82)	−2.19 (0.79)	−2.07 (0.79)	−2.16 (0.79)	−2.20 (0.80)	−2.19 (0.79)	−2.05 (0.88)	−2.42 (0.87)	−1.90 (0.81)	−2.11 (0.81)	−2.37 (0.84)
Environmental axis	Wald	53.20	0.13	0.56	5.30	2.94	11.75	0.08	96.98	239.89	366.50	421.70
	P	<0.001***	0.713	0.456	0.021*	0.087	<0.001***	0.783	<0.001***	<0.001***	<0.001***	<0.001***
	parameter estimate (S.E.)	0.31 (0.04)	−0.02 (0.04)	−0.03 (0.03)	0.08 (0.03)	0.07 (0.04)	0.13 (0.04)	0.01 (0.04)	0.36 (0.04)	0.53 (0.03)	0.67 (0.04)	0.73 (0.04)
Inbreeding * Environmental axis	Wald	0.24	0.17	1.30	0.32	1.37	2.31	0.11	5.97	0.39	0.54	2.06
	P	0.622	0.679	0.255	0.571	0.241	0.128	0.742	0.015*	0.533	0.462	0.152
	parameter estimate (S.E.)	−0.82 (1.66)	−0.64 (1.56)	−1.73 (1.52)	−0.85 (1.49)	−1.85 (1.58)	2.48 (1.63)	0.57(1.72)	3.98 (1.63)	−0.99 (1.58)	1.26 (1.71)	2.77 (1.93)
*Model:* Sector +Clutch size+Egg laying date+…			+year	+year	+year	+year	+year	“egg laying date” excluded from the model				

Generalised linear mixed model with Poisson errors, logarithm link and parental identity fitted as random effects.

* : P<0.05;

** : P<0.01;

*** : P<0.001.

**Figure 2:**
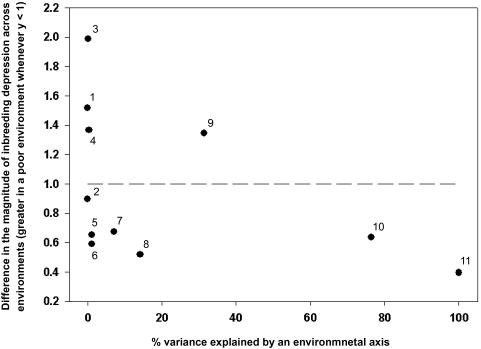
Difference in the magnitude of inbreeding depression in recruitment across environmental axes in the great tit. The difference in the magnitude of inbreeding depression is here defined as the difference in inbreeding depression between good and bad environments; each data point refers to one environmental axis. For all cases where *y*<1, the point estimate for inbreeding depression was larger in the poor environment relative to the good one. Where *y*>1, inbreeding depression was less severe in a bad environment than in a good environment. The numbering of each data point refers to the numbering in table 1 and represents the following environmental axes: (1) yearly population density of breeding events, (2) local oak density, (3) female parental age, (4) male parental age, (5) local population density of breeding events, (6) nestbox distance from forest edge, (7) lag between caterpillar peak and hatching peak, (8) fledging mass, (9) winter beech mast abundance, (10) phenotypic coefficient of variation in recruitment, and (11) yearly quality in recruitment.

It is not expected to find an interaction between inbreeding and the environment in environmental axes that do not influence fitness, as these effectively simply split the dataset in two. Although the strength of inbreeding depression varied substantially across environmental axes, this variation was mainly observed in those environmental axes that explained little of the variation in recruitment. In contrast, we would predict consistently larger inbreeding depression in poor environments the closer the environmental axis was related to fitness. In line with this expectation, we found that inbreeding depression in poor environments (relative to good environments) was larger the more closely the environmental axis was related to fitness (Spearman rank correlation: *r*_S_ −0.699, t approximation = 2.93, d.f. = 9, n = 11, p = 0.017; Figure 2). This result suggests that the difference between inbred and outbred individuals depends not just on the difference in the environment, but also on the relevance of that environment for fitness.

## Discussion

We analysed a large data set on inbreeding and fitness in a wild great tit population; although both inbreeding, and seven of the eleven environmental axes, significantly affected survival at the recruitment stage, we found little statistical support for interactions between inbreeding and environmental quality when each environmental axis was tested independently. However, we did find a significant trend for the size of this interaction to be larger the more closely the environmental axis was related to overall fitness. This suggests that there should be particular scope for an interactive effect between inbreeding and the environment when environments and phenotypic traits highly linked to fitness are used; repeating a similar analysis on other datasets where plant and animal inbreeding have been investigated would be worthwhile. We emphasise the importance of using environmental variables that have an effect on individual fitness when testing any type of genotype by environment interaction with respect to a fitness character; failure to do so will result in testing the effect of particular genotypes on fitness, but not its interaction with the environment.

The low statistical power of our analyses suggests that testing for I×E interactions will be quite difficult in single studies of populations like ours, where inbreeding is relatively rare. Despite more than 40 years' data, and in excess of 4000 fully characterised reproductive events, we were able to identify only 58 inbreeding events at *f*≥0.125. In contrast, insular populations may offer much better opportunities for testing I×E interactions. Often, the rate of close inbreeding will be much higher because of the relatively small population size, and the insular nature of the population also facilitates the estimation of inbreeding coefficients from longer pedigrees [e.g. 22].

In their recent review of experimental studies investigating inbreeding by environmental interactions, Armbruster and Reed [4] suggest that the detection of I×E interactions may be difficult because fitness has a lower bound at zero. Hence, in increasingly severe environmental conditions, the expression of inbreeding depression may be constrained when units of fitness (such as number of recruits) are bounded by zero. As in our study, many studies that found no evidence of I×E interactions report fitness values of the inbred treatment in the stressful environment that approach zero (see for example Norman et al. [23], Hauser and Loeschcke [24] and Armbruster and Reed [4]). As fitness gets closer and closer to zero, the effect of measurement error, and stochasticity in the environment, will get proportionately larger, which will make the detection of significant effects much harder. One extension to this reasoning is the suggestion that inbreeding depression should be most pronounced in environmental conditions that enhance the variability of fitness traits. This idea was tested on *Brassica rapa* by Waller and colleagues (D. Waller, pers. comm.), and as in their study, we find the same trend suggesting that inbreeding depression may be constrained by phenotypic variability (see Table 1, Figure 2), for example by being bounded by zero. Investigating environmental conditions that limit or enhance phenotypic variability opens the way to an interesting alternative to the traditional testing of inbreeding along a gradient of “stressful” environments, and deserves exploration in other populations.

While testing for the ubiquity of inbreeding by environment interactions, it must be borne in mind that the effect size of any such interaction will also depend on the proportion of unconditional versus conditional recessive alleles in the population. If all recessive alleles in the population are phenotypically expressed to the same degree independently of environmental conditions (i.e. unconditional alleles), no inbreeding by environment interaction would be observed. Clearly, our results show that there is scope for I*E interactions, particularly in later life history stages as those are expected to be more prone to the expression of conditional, milder recessive alleles which are difficult to purge, and contrast with unconditional lethals, acting in early life history stages [25], [26].

The conclusion from our study with respect to the existence of I×E interactions is mixed. Statistical analysis provides no clear support for the presence of single I×E interactions, yet estimates of the size of any such effect are large (and with large confidence intervals), as revealed by the analysis of lethal equivalents in the two environments. Moreover, inbreeding depression worsens in poor environments that strongly affect overall fitness. Hence, it would be unwise to conclude that such effects are not present. While it will be difficult to either demonstrate or rule out the existence of such effects from single studies of wild populations where inbreeding is rare, it is important that such estimates as do exist are published because this affords the possibility of synthesising across studies. Although it is important to estimate the size of any I×E interactions in wild populations, individual studies may have a limited capacity for uncovering detailed patterns of environment-dependent expression of deleterious recessives. Controlled experiments in a semi-natural environments [e.g. 11], [12], [27], using individuals with known pedigree, genotypes and levels of inbreeding might provide the best solution to yield quantitatively reliable estimates of the effect size of interactions between inbreeding in the environment. Indeed, such an approach would reconcile the need for appropriately large sample size with the relevance of the environment in which I*E interactions are measured to the ecology and evolution of the species studied.

## Materials and methods

### Study Population and Pedigree Building

The Wytham Woods (Oxfordshire, U.K.) great tit (*Parus major*) population has been monitored since 1947 [28]. The current population study involves 1020 nestboxes scattered across the single woodland at variable densities, which have been in position since 1961. Nestlings are ringed individually with metal rings on day 15 after hatching, and parents are caught at the nestbox while feeding young. Adult identities are recorded through their ring number, and immigrant individuals (i.e. not born in Wytham Woods) are ringed; the rates of immigration to the population are quite high, as 47% of females and 41% of males breeding in any year within Wytham are born outside the woods [29]. We used data collected from this procedure to build a pedigree, which included all great tit breeding events recorded in Wytham and its vicinity between 1958 and 2004. Unknown breeding parents (e.g. when one member of a pair was not captured) were assigned a unique identification number specific to the breeding event they took part in; breeding events that were subject to egg or offspring cross-fostering where the biological parents could not be identified were removed from the pedigree. Their descendents, however, were included in the pedigree, and were assumed to have unknown and unrelated parents.

The pedigree linked 71 008 individual great tits, with a mean pedigree depth of 7.7 generations (median = 4; see [14]). Inbreeding coefficients were estimated from pedigree data using Pedigree Viewer version 5.2 (available free at http://www-personal.une.edu.au/∼bkinghor/pedigree.htm). Our final dataset included 4523 breeding events between 1964 to 2004 where both parents and at least one grandparent were known. Restricting the dataset in this way allowed us to exclude breeding events where we would not be able to identify cases of very close inbreeding (*f*≥0.125) [13], [14], [30]. Because more must be known about more ancestors in order to identify cases of less close inbreeding [see 14], [30], which would greatly reduce the sample size available for analysis, we decided to fit inbreeding as a continuous variable with three levels of inbreeding: *f* = 0.0, *f* = 0.125, and *f* = 0.25. Breeding events that had been subject to manipulations such as cross-fostering, brood enlargement or reductions (potentially influencing survival of offspring) were excluded from our analysis. More details on pedigree building in an inbreeding context, the calculation of inbreeding coefficients and sample sizes can be found in Szulkin et al. [14].

We could not identify extra-pair offspring in our dataset; given that in this population, estimates of the proportion of extra-pair young are of the order of 15% [31, Patrick, unpublished data], this might lead to a false categorization of some outbred offspring as inbred. Overall however, although we might underestimate the effect size of inbreeding depression, the downward bias should not be substantial, and as far as we are aware, any such effect will be independent of the environmental indices we used to test for I×E interaction. All calculations were made at a brood level in order to take into account the non-independence of offspring from the same breeding event. Over 41 years of long-term study, 4523 breeding events were recorded where both parents and at least one grandparent were known; we identified 45 breeding events where offspring were inbred at *f* = 0.25 and 13 at *f* = 0.125.

### Environmental Axes

Eleven measures of the quality of the environment, referred to as “environmental axes”, were used to describe the environment experienced by great tit offspring either while in the nest or subsequently up to recruitment. Those encompassed both measures of “static” environmental heterogeneity, such as the relative position of the breeding site relative to the habitat edge, or oak richness within 50 meters of the nestbox, as well as dynamic measures, such as population density, or annual synchronisation with caterpillar peaks (main food source for nestlings) etc. All eleven axes and predictions as to their effect on recruitment are presented in Table 1.

We used *N_R_*, the number of offspring recruited per breeding event, as an overall measure of fitness for any given breeding event. We then tested the relationship between *N_R_* and the eleven environmental cues suggested to impact on offspring fitness. These would vary in terms of their mode of action, as they target in a unique way the breeding event, nestbox or cohort (Table 1). The method used to determine whether each breeding event, nestbox or cohort experienced a good or poor environment is as follows. For five environmental cues acting at a cohort (yearly) level (Table 1, nrs. 1, 7, 8, 10, 11), we calculated the corresponding mean annual environmental values *E_i_* for each year *i*, where “*i*” ranges from 1 to 41 (i.e. the number of breeding years in the dataset); we then calculated the median value *M_E_* of all values of *E_i_*. All cases where *E_i_>M_E_* and *E_i_ ≤M_E_* were defined as “good” and “bad” years in terms of a particular environmental axis, respectively. Analyses at the nestbox and breeding event level were made in similar fashion: environmental cues acting at a nestbox level (E*_b_*) were used to calculated a nestbox-based median value of environmental cues; all cases where *E_b_>M_E_* and *E_b_ ≤M_E_* were defined as “good” and “bad” environments (nestboxes) in terms of a given environmental axis, respectively. In cases where the environmental cues were measured uniquely for each breeding event (E*_ev_*), we used all breeding events to calculate a median value of that particular environmental cue. Cases where E*_ev_*>*M_E_* and E*_ev_ ≤M_E_* were defined as “good” and “bad” environments for a given breeding event. Finally, yearly quality in beech mast (important food source to great tits in the winter when available) was scored as either 0 or 1 [based on 32], where 0 is equivalent to poor winter beech mast years, and 1 reflects large amount of food in the winter. This environmental axis thus splits the dataset into two unequal categories where 29 and 12 years were categorised as poor and good years in terms of beech mast abundance, respectively.

If inbreeding depression acts with increasing strength in poor environmental conditions, we expect to find a significant interaction between the inbreeding coefficient and the measure of the environment on a measure of fitness. We tested the interactive effect of inbreeding and each of the eleven environmental axes on recruitment using generalised linear mixed models with Poisson errors, a logarithm link and parental identity fitted as random effects in all models. An estimate of the dispersion parameter was used to control for overdispersion. Models were fitted with a range of fixed effects that previous work on the population has shown to be important in explaining fitness variation among individuals, such as forest sector, egg laying date, clutch size or year where the breeding event took place. These variables were chosen so as not to limit the breeding dataset.

We further tested the strength of inbreeding depression in good and poor environments using 11 environmental axes that explained a varying amount of variance in fitness (Table 1). We predicted that inbreeding depression would be greatest in poor environments of those environmental axes that have the greatest influence on fitness. To avoid pseudo-replication in estimates of the amount of variance explained by each environmental axis on fitness, we performed our analyses using either annual (cohort), nestbox-specific or breeding event specific values of environmental quality, depending on the characteristics of each axis (Table 1). A linear regression of annual recruitment explained 100% of the variance in annual recruitment (by definition), while the remaining 10 environmental axes explained 0–76.4% of the variance in recruitment (Table 1). The amount of variance explained by the environmental axes was subsequently plotted against the magnitude of difference in inbreeding depression, calculated as:

[(mean recruitment of *f* = 0.25 broods in a poor environment)/(mean recruitment of *f* = 0.25 broods in a good environment)]/[(mean recruitment of *f* = 0.0 broods in a poor environment)/(mean recruitment of *f* = 0.0 broods in a good environment)]

Thus, if inbred and outbred individuals are equally affected by the environment, we obtain a value of 1. The ratio is smaller than 1 in the case where inbred individuals do worse in a poor environment than outbred individuals. Two points are worth making here. First, while the proportion variance in recruitment explained by the annual mean recruitment is, by definition, unity, it is legitimate to use this point in the analysis because under the null hypothesis that there is no relationship between the size of inbreeding depression, and the effect of the environment, this point still provides useful data. Second, even when individual estimates of I×E interaction are non-significant, the size of these estimates can still be used as data points for analysis, as here. Analysis was carried out using Genstat Version 8.1 (VSN international Ltd).

### Lethal Equivalents

The decline in fitness due to inbreeding can be quantified in a standardized way using lethal equivalents, expressed in units where one lethal equivalent (B) would cause one death in a homozygote [33]. We estimated the number of lethal equivalents at the fledging and recruitment stage as
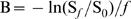
where S*_f_* is the probability of survival at inbreeding level *f* = 0.25 and S_0 _is the probability of survival at inbreeding level *f* = 0. A standard error for the number of lethal equivalents can be calculated [see 1], but the standard errors returned by this method were unrealistically small, while the analysis of I×E interactions provides reliable information as to the confidence in the size of the lethal equivalent estimates in the two environments. We thus present merely the point estimate for the number of lethal equivalents, in which for a diploid genome it is twice the rate of increase in mortality caused by inbreeding, and is thus equivalent to 2B.
